# Attention to Stimuli of Learned versus Innate Biological Value Relies on Separate Neural Systems

**DOI:** 10.1523/JNEUROSCI.0925-22.2022

**Published:** 2022-12-07

**Authors:** Peter M. Kaskan, Mark A. Nicholas, Aaron M. Dean, Elisabeth A. Murray

**Affiliations:** ^1^Leo M. Davidoff Department of Neurological Surgery, Albert Einstein College of Medicine, Bronx, New York 10461; ^2^Section on Neurobiology of Learning and Memory, Laboratory of Neuropsychology, National Institute of Mental Health, National Institutes of Health, Bethesda, Maryland 20892

**Keywords:** amygdala, orienting, attentional capture, pupil diameter, stimulus-reward learning, macaque

## Abstract

The neural bases of attention, a set of neural processes that promote behavioral selection, is a subject of intense investigation. In humans, rewarded cues influence attention, even when those cues are irrelevant to the current task. Because the amygdala plays a role in reward processing, and the activity of amygdala neurons has been linked to spatial attention, we reasoned that the amygdala may be essential for attending to rewarded images. To test this possibility, we used an attentional capture task, which provides a quantitative measure of attentional bias. Specifically, we compared reaction times (RTs) of adult male rhesus monkeys with bilateral amygdala lesions and unoperated controls as they made a saccade away from a high- or low-value rewarded image to a peripheral target. We predicted that: (1) RTs will be longer for high- compared with low-value images, revealing attentional capture by rewarded stimuli; and (2) relative to controls, monkeys with amygdala lesions would exhibit shorter RT for high-value images. For comparison, we assessed the same groups of monkeys for attentional capture by images of predators and conspecifics, categories thought to have innate biological value. In performing the attentional capture task, all monkeys were slowed more by high-value relative to low-value rewarded images. Contrary to our prediction, amygdala lesions failed to disrupt this effect. When presented with images of predators and conspecifics, however, monkeys with amygdala lesions showed significantly diminished attentional capture relative to controls. Thus, separate neural pathways are responsible for allocating attention to stimuli with learned versus innate value.

**SIGNIFICANCE STATEMENT** Valuable objects attract attention. The amygdala is known to contribute to reward processing and the encoding of object reward value. We therefore examined whether the amygdala is necessary for allocating attention to rewarded objects. For comparison, we assessed the amygdala's contribution to attending to objects with innate biological value: predators and conspecifics. We found that the macaque amygdala is necessary for directing attention to images with innate biological value, but not for directing attention to recently learned reward-predictive images. These findings indicate that the amygdala makes selective contributions to attending to valuable objects. The data are relevant to mental health disorders, such as social anxiety disorders and small animal phobias, that arise from biased attention to select categories of objects.

## Introduction

Attention regulates the deployment of cognitive resources to behaviorally relevant items, enhancing the processing of some items at the expense of others. In the context of visual sensory processing, stimuli may attract attention by their physical salience (e.g., size, color) or because a subject is instructed to implement a rule based on a particular feature or class of features. These two forms of attention are typically called “bottom-up” and “top-down,” respectively (Desimone and Duncan, 1995). More recently, a number of frameworks for understanding attention have been described (Awh et al., 2012; Nobre and Mesulam, 2014; Le Pelley et al., 2016; Moore and Zirnsak, 2017; Krauzlis et al., 2021). These frameworks highlight the multiple and sometimes contradictory sources of attentional bias.

In humans, attention is biased toward threatening cues, such as predators, and toward social cues, such as faces (Mogg and Bradley, 1999; Pourtois et al., 2004; Rosler et al., 2005; Dahl et al., 2009). In addition, stimuli associated with reward can slow (Della Libera and Chelazzi, 2009; Rutherford et al., 2010; Anderson et al., 2011b) or speed (Hickey et al., 2014) visual search in a spatially specific manner and can also interfere with cognitive performance like that required for the Stroop task (Krebs et al., 2011). Although there is a paucity of work in nonhuman primates using standard measures of attentional bias, such as reaction time (RT) (e.g., Landman et al., 2014; Dal Monte et al., 2015), there are several other behavioral indices that strongly suggest attentional bias toward socially informative cues. For example, macaques spend more time viewing faces of conspecifics relative to objects, prioritize initial gaze to faces over objects (Taubert et al., 2017), and will forfeit or delay reward to view images of conspecifics (Deaner et al., 2005; Rudebeck et al., 2006), prioritizing dominant individuals and female perinea for longer viewing. In the context of an approach-avoidance conflict task, macaques also forfeit reward when in the presence of certain predators (Amaral et al., 2003; Izquierdo and Murray, 2004; Machado et al., 2009; Charbonneau et al., 2022). Finally, visual attention to objects and object features in macaques is affected by the reward history of those objects or features (Jagadeesh et al., 2001; Kobayashi et al., 2002; Peck et al., 2009; Roe et al., 2012; Stănişor et al., 2013; Xie et al., 2018). Thus, visual attention in both humans and nonhuman primates is profoundly affected by stimuli in three categories: predators, conspecifics, and stimuli associated with reward.

The phenomenon whereby stimuli interfere with ongoing cognitive processes, even when those stimuli are irrelevant to the task at hand, is known as attentional capture, and is typically evidenced by a slowing of RT to achieve a goal relative to baseline conditions. Consistent with its role in processing social cues related to facial expressions (Adolphs et al., 1994; Morris et al., 1996; Anderson and Phelps, 2001; Fried et al., 2002; Adams et al., 2003; Rutishauser et al., 2011), the amygdala makes a causal contribution to attentional capture by images of faces (Dal Monte et al., 2015). There is also a literature on attentional capture by rewarded stimuli in humans (Anderson et al., 2011b; Chelazzi et al., 2013). Given that the amygdala contributes to reward learning (Baxter and Murray, 2002; Hampton et al., 2007), and that the activity of amygdala neurons is correlated with visual spatial attention for stimuli that have been associated with reward (Peck et al., 2013), we wondered whether the amygdala would be essential for attentional capture by rewarded stimuli, as it is for social stimuli. Accordingly, we examined attentional capture by recently learned reward-predictive visual stimuli in monkeys with amygdala lesions (*N* = 3) and unoperated controls (*N* = 3). For comparison, the same groups of monkeys were assessed for attentional capture by naturalistic images of predators and conspecifics, relative to neutral images. We predicted that the amygdala would play an essential role in attending to all three categories of stimuli.

## Materials and Methods

### Subjects

Six adult male rhesus monkeys (*Macaca mulatta*) served as subjects in this study. Monkeys were obtained from a domestic breeding colony; they were born and raised in outdoor corrals, with access to shelter from the elements, in groups ranging from ∼10 to 50 individuals. Veterinarians and technicians trained in macaque husbandry were on site to monitor the monkeys' health and well-being throughout the rearing period. When the monkeys were ∼3-5 years of age, they were transported to the National Institutes of Health Animal Center to undergo quarantine and then ultimately brought to our animal facility. Upon transfer to our facility, animals were housed and enriched in a manner consistent with the *Guide for the Care and Use of Laboratory animals*.

Three monkeys had received bilateral excitotoxic lesions of the amygdala (Lesion 1 [L1], L2, and L3); 3 unoperated monkeys served as controls (Control 1 [C1], C2, and C3). At the beginning of the experiment, monkeys with amygdala lesions weighed, on average, 10.3 kg (range: 8.6-12.2 kg) and controls weighed 8.9 kg (range: 7.5-9.9). There was no group difference on this measure. As for age, monkeys with amygdala lesions averaged 11.5 years of age (range 7.8-17.1 years) and controls averaged 7.1 years of age (range 5.7-9.7 years) at the beginning of the study. There was no group difference on this measure. Before the present study, all monkeys had extensive experience with stimulus-reward learning in computer-run test apparatuses. Monkeys were individually housed and kept on a 12 h light-dark cycle with scheduled access to food, and controlled access to water during testing. All procedures were conducted in accordance with the *Guide for the care and use of laboratory animals* and were approved by the National Institute of Mental Health Animal Care and Use Committee.

### Surgical procedures

Ibotenate injections were placed stereotaxically throughout the amygdala based on coordinates from a preoperatively acquired MRI scan. Details of the procedure have been reported previously (Izquierdo and Murray, 2007; Dal Monte et al., 2015). The amygdala injections were conducted in two stages, 2 weeks apart, and were balanced for hemisphere of first operation.

All monkeys had a head post surgically implanted to allow for head fixation and accurate video eye tracking. Monkeys with amygdala lesions were given at least 60 d of recovery before head post implantation. A minimum of 30 d intervened between the head post implantation and the initiation of training.

### Lesion assessment

**Figure 1.**
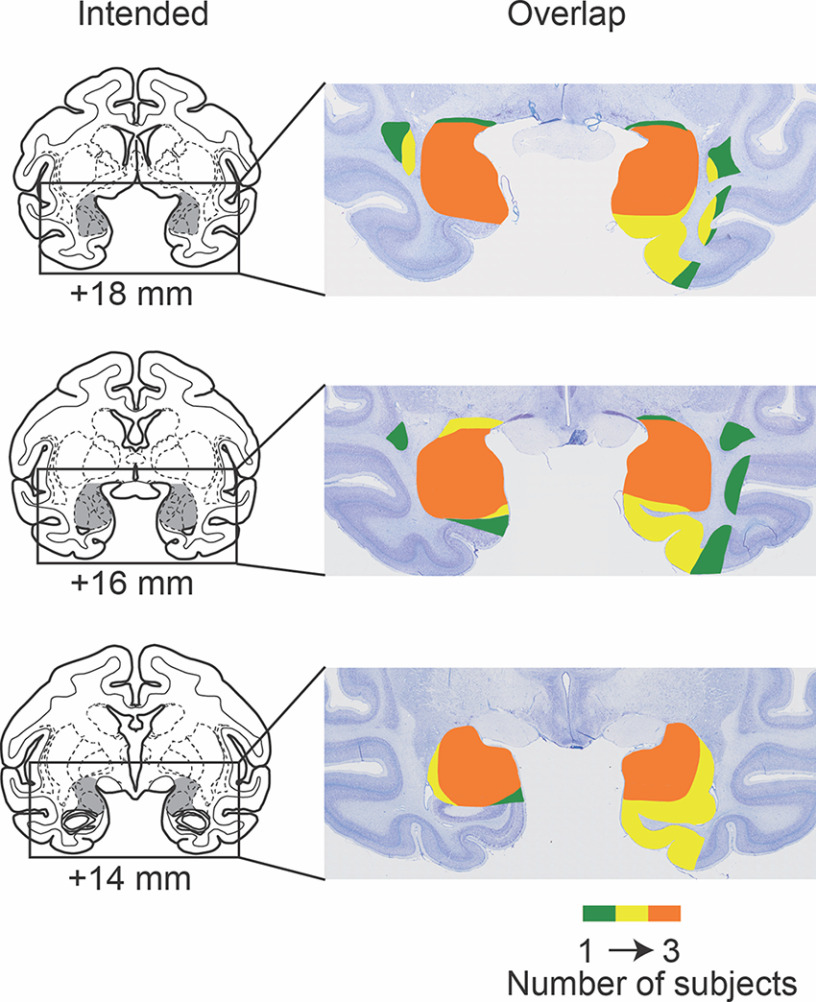
Reconstruction of amygdala lesions. Left column, Coronal sections from a standard rhesus monkey brain depicting the extent of the intended bilateral amygdala lesion (shaded regions). Numerals indicate the distance (in millimeters) from the interaural plane (0). Right column, The location and extent of the excitotoxic lesions in the 3 monkeys in the lesion group plotted on photomicrographs of Nissl-stained coronal sections from an unoperated rhesus monkey. Sections were selected from anterior-posterior levels to match those shown on the left. Colors represent the extent of lesion overlap in monkeys in the amygdala lesion group.

**Figure 2.**
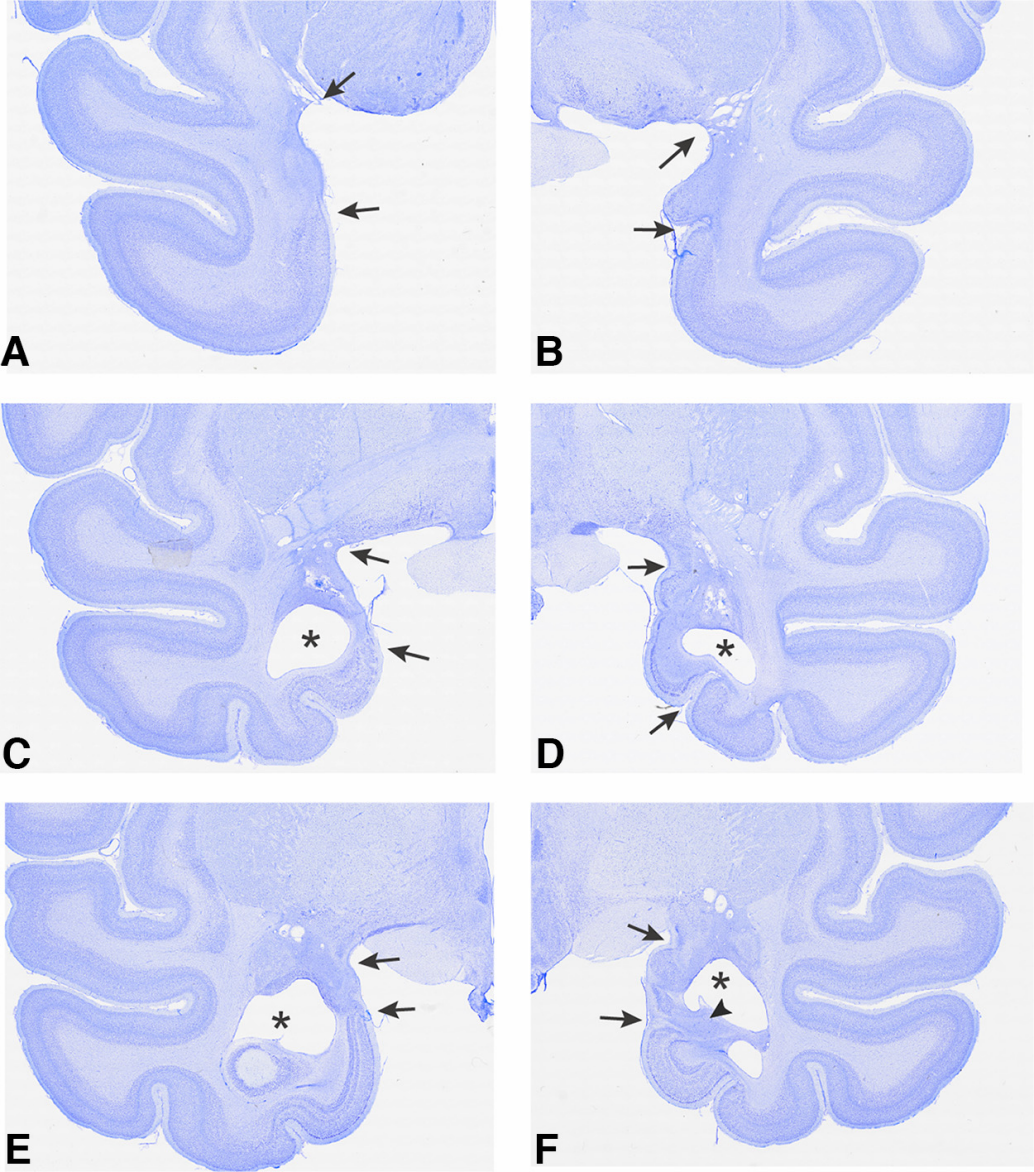
Photomicrographs of Nissl-stained coronal sections from 1 monkey (L2) in the amygdala lesion group. Sections are ∼19 (***A***,***B***), 16 (***C***,***D***), and 14 (***E***,***F***) mm anterior to the interaural plane (0). The left and right images for each pair (e.g., ***A*** and ***B***) are from different sections because of asymmetry in the plane of section. Small black arrows indicate boundaries of the lesion at the pial surface. Asterisks indicate the expansion of the temporal horn of the lateral ventricle into the space created by cell loss in the amygdala. ***F***, Arrowhead indicates cell loss in the anterior tip of the right hippocampus.

Lesions were quantitatively assessed using postoperative MRI. Amygdala damage was estimated using T2-weighted scans acquired within 10 d of the operation (Basile et al., 2017). For each monkey, scan slices were matched to drawings of a standard rhesus monkey brain at 1 mm increments; and the location of white hypersignal, indicative of edema at the injection sites, was plotted onto the standard sections. Using these plots, the percent damage to the amygdala could be estimated. Figure 1 illustrates the extent of the lesion overlap in the 3 subjects with amygdala lesions, and the photomicrographs in Figure 2 document the cell loss in the amygdala and resulting atrophy in 1 monkey (L2). Based on the T2-weighted scans, the amygdala lesions in all 3 operated monkeys appeared to be complete, or nearly so. Two monkeys sustained inadvertent damage to the entorhinal cortex, perirhinal cortex, and anterior hippocampus in one hemisphere.

### Apparatus and materials

All training was performed while monkeys were seated in custom-built primate chairs and testing booths. While under head restraint, monkeys sat with their head 57 cm from a 19 inch color LCD monitor. Visual stimuli selected from the Internet or the authors' personal collections were presented against a 50% gray background. Images were scaled and cropped to 300 × 300 pixels using Adobe Photoshop (RRID:SCR_014199) and balanced for luminance using the SHINE toolbox (Willenbockel et al., 2010) in MATLAB (The MathWorks; RRID:SCR_001622). In addition, the images for Experiment 2 were phase-scrambled to create a control image group in which second-order statistics were preserved but higher-order statistics of each image were destroyed. Eye movements were monitored using an eye-tracking system (Arrington ViewPoint). MonkeyLogic (Asaad and Eskandar, 2008) was used to both record eye positions and present stimuli on the monitor. The monitor screen was the sole source of light within the testing booths and remained constant across all experimental sessions.

For Experiment 1, three images of basic shapes and a set of 32 images of colored objects selected from the Internet were used for preliminary training. A separate set of 90 images of colored objects was used for the main task. No more than three images of any given object type (i.e., shoes, pencils, coffee cups, etc.) were used, and all images were novel at the beginning of the experiment.

Visual stimuli for Experiment 2 consisted of a set of 3000 colored images. For the main task, we used a total of 1320 images of neutral objects, 90 images of conspecifics (female perinea), and 90 images of predators (snake, spider, crocodile, or alligator). As indicated above, each of the intact images was phase-scrambled to create a scrambled control image. Thus, the entire stimulus set comprised 1500 intact images and 1500 scrambled images.

### Overview of experimental design

**Figure 3.**
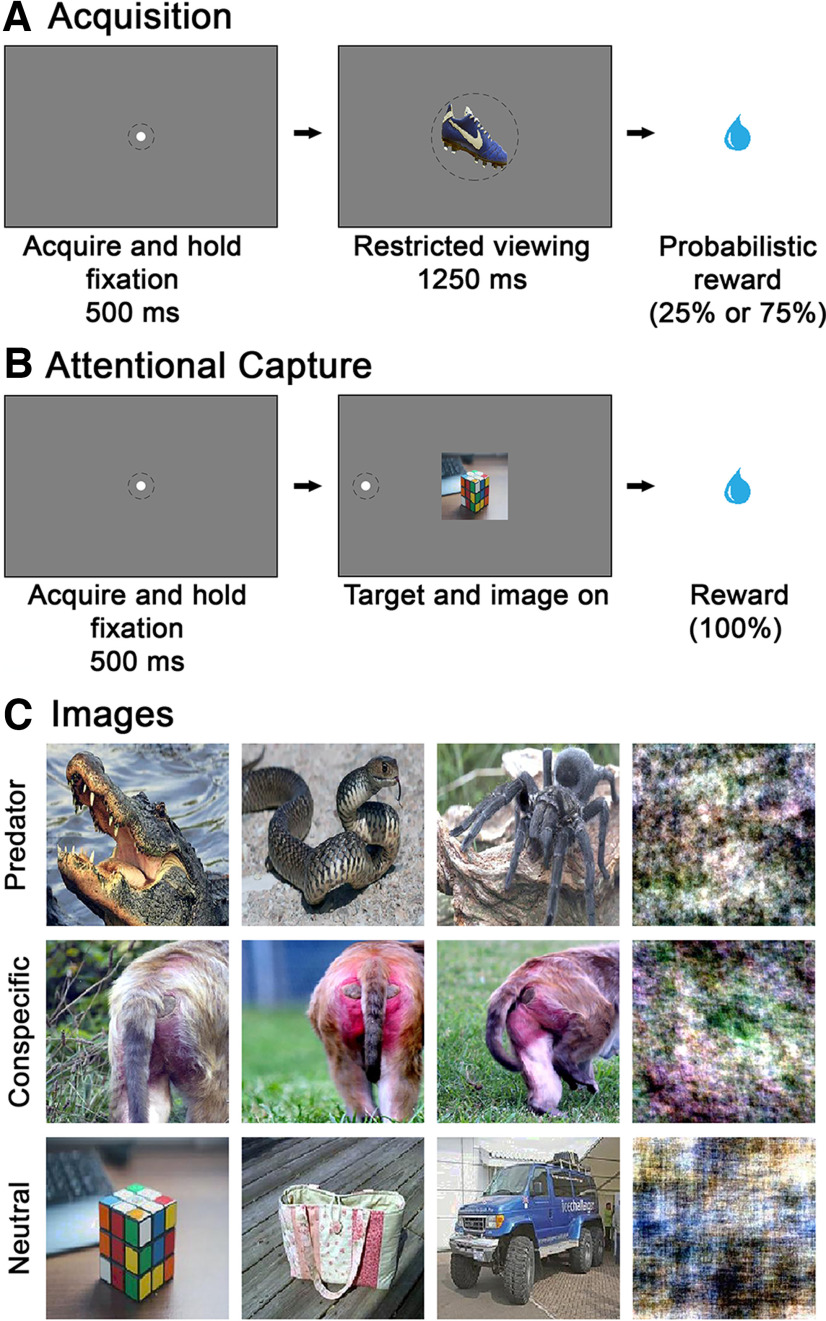
Task design and visual stimuli. ***A***, Acquisition of image-reward associations in Experiment 1. Monkeys faced a monitor screen on which they could initiate trials by maintaining their gaze within 3 DVA around a central fixation spot. After monkeys maintained gaze for 500 ms, the fixation spot was replaced with a single image. The monkey was required to maintain gaze within 6 DVA around the image for an additional 1250 ms to obtain a water reward based on the predetermined reward probability for that image (high or low). ***B***, Attentional capture task used in Experiments 1 and 2. Monkeys could initiate trials by maintaining gaze on the fixation spot. After completion of the fixation requirement, both a centrally placed image and a peripheral target (12 DVA to left or right of center) appeared simultaneously. The location of the peripheral target (left or right) followed a random order. The monkey was required to saccade away from the central image to the peripheral target to obtain a water reward. ***C***, Example images used in the attentional capture task in Experiment 2. Naturalistic images of predators and conspecifics comprised 6% of the 200 novel cues on a given day (3% predator and 3% conspecific). Forty-four percent of the images were neutral objects, and the remaining 50% were the matched phase-scrambled images. Examples of phase-scrambled images (right column) are shown adjacent to the intact image from which they were derived.

We conducted two separate experiments. Experiment 1 assessed the extent to which recently rewarded stimuli captured attention. Because both reward value and strength of learning seemed likely to influence the degree of bias, we systematically varied both factors during Acquisition trials. To manipulate reward value, we trained monkeys on a set of stimulus-reward associations involving either a high (75%) or low (25%) probability of reward (Fig. 3*A*). We manipulated the degree of learning by presenting the same stimuli over a series of days. These images were then used in a standard paradigm to assess attentional capture (Fig. 3*B*). On each day, 12 images were shown: 6 high probability and 6 low probability of reward images. Two of the 12 images (one high and one low probability of reward image) were presented every day and constituted overtrained images. Each day, a novel image set (one high- and one low-probability of reward image) was introduced and the image set that had been in rotation longest was removed. Ninety images were shown in total (12 images on the first day plus two substitutions per day for the subsequent 39 d: 12 + 78 = 90 images). In this rolling design, the reward probability assigned to individual images (high or low) was experienced for 5 consecutive days.

In the Main task, we trained monkeys on three blocks of trials in each daily session. In the first block, monkeys had the opportunity to acquire stimulus-reward associations (Acquisition trials). The second block was an instructional block to provide a transition to the third block (Attentional Capture trials) in which monkeys were assessed for attentional capture by recently rewarded stimuli.

Experiment 2 assessed the influence of innately valuable images on attentional capture. For this, we used stimulus classes known to elicit visual interest in rhesus macaques (e.g., conspecific [social] cues, predators) without any formal training (Deaner et al., 2005; Rudebeck et al., 2006).

### Preliminary training

Monkeys were first trained to fix their gaze on a spot on the monitor screen using standard positive reinforcement procedures. They were then trained on the structure and response requirements of both Acquisition trials and Attentional Capture trials. In preliminary training stages, monkeys could initiate trials by maintaining their gaze within a 3 degrees of visual angle (DVA) circular window centered around the fixation spot. If gaze was successfully maintained, a randomly selected image from the preliminary training set, similar to those used in the main experiment, would replace the central fixation spot and the monkey had to maintain its gaze within a circular window of 6 DVA around the image to obtain a water reward. Over the course of several weeks, the duration of central fixation both before and after image onset that monkeys were required to perform was increased to allow for adequate recording of pupil responses. This procedure served as the basis for the Acquisition trials.

After monkeys were reliably holding their gaze on the central fixation spot for 1 s and holding their gaze on the image for 2 s, they were trained on an oculomotor saccade task. Monkeys were required to initiate the trial under the same conditions as before. Now, however, after successful initiation of a trial, both a centrally located image and a peripheral target appeared simultaneously. The peripheral target was located 12 DVA from the central image on either the right or left side of the monitor. The monkey could obtain a reward by making a saccade away from the central image, acquiring the peripheral target, and maintaining gaze within a 3 DVA circular window about the peripheral target. The time allowed to make a saccade to the peripheral target was decreased over several weeks to encourage monkeys to promptly saccade to the target. Monkeys were trained until they were able to acquire the peripheral target within 200 ms; in the experiments, this requirement was relaxed to 300 ms and then 1000 ms so as to not constrain RTs and potentially limit the attentional capture effect. This procedure served as the basis for the Attentional Capture trials; RT, defined below under Experiment 1 Main task, was our dependent measure.

Once monkeys had mastered the above procedures, separately, they were trained on both tasks within each day. Fixation gaze and image gaze times were reduced to 500 ms and 1250 ms, respectively, and monkeys were trained for several weeks until the percentage of correct trials across both tasks was >70%.

### Experiment 1: attentional capture by rewarded images

#### Main task

The task was organized into three blocks: Acquisition, Instructional, and Attentional Capture. These three blocks were administered one after the other and given in the same order each day.

During the Acquisition block, monkeys were given the opportunity to learn image reward associations. Monkeys initiated a trial by maintaining their gaze on a white central fixation spot on a monitor for 500 ms. After the fixation requirement was fulfilled, an image replaced the central fixation spot, and monkeys were required to maintain their gaze within 6 DVA around the image for an additional 1250 ms to obtain a water reward (0.4 ml) according to the probability (high or low) assigned to that stimulus (Fig. 3*A*). Any failure to hold fixation was considered an error, and the trial was terminated. For the 12 images each day, individual images were presented in a pseudorandom order until monkeys completed 20 trials per image resulting in 240 correct Acquisition trials. All images were counterbalanced across the 6 monkeys, and all monkeys had unique overtrained images. Intertrial intervals were randomly selected from a uniform distribution ranging from 3 to 5 s. If monkeys required over 90 min to complete the block of Acquisition trials, or if they did not complete at least 50 trials within the first 100 trials, the experiment was stopped and monkeys were presented with the same images on the following day. Of the 40 d of the experiment, monkeys repeated 1.83 ± 2.56 (mean ± SD) days. Upon completion, monkeys were advanced to the Instructional block.

The Instructional block started with a 30-s unfilled interval intended to alert monkeys to the impending change in task demands. Monkeys were then given 10 practice Attentional Capture trials, all conducted with a single image dedicated to this block. Monkeys were rewarded on every trial in which they successfully acquired the peripheral target.

For trials in the Attentional Capture block, monkeys initiated trials in the same manner as for Acquisition trials. Unlike Acquisition trials, however, the central fixation spot was replaced by an image previously seen during the Acquisition block together with a peripheral target (Fig. 3*B*). The peripheral target was identical to the central fixation spot and was presented randomly on either the left or the right side (12 DVA) of the centrally located image. Monkeys were required to saccade to the peripheral target and maintain their gaze for 100 ms within a 3 DVA circular window to obtain a water reward (0.4 ml). Monkeys were presented with individual images in a pseudorandom order until they correctly completed 10 Attentional Capture trials for each of the 12 images resulting in 120 completed Attentional Capture trials per session. RT was defined as the time between the onset of the peripheral target and the initiation of monkeys' saccades, taken to be the time the monkeys' gaze deviated from the circular 3 DVA central fixation window. Monkeys were trained 5 d per week for a total of 40 d.

### Experiment 2: attentional capture by conspecific and predator images

#### Main task

Monkeys were tested on the same Attentional Capture task using naturalistic images of conspecifics (female perinea) and predators (e.g., snakes, spiders, alligators, crocodiles), in addition to images of neutral objects (Fig. 3*C*). Each daily session consisted of a single 200-trial Attentional Capture block in which monkeys were tested on 200 novel images. Images were 3% social cues (6 per day; 90 images in total), 3% predators (6 per day; 90 images in total), 44% neutral objects (88 per day; 1320 images in total), and 50% scrambled (100 per day; 1500 images in total), presented in pseudorandom order until monkeys successfully completed one attentional capture trial per image. Monkeys received 0.5 ml of water on completion of each trial. Monkeys were tested on this task 5 d per week for a total of 15 d.

### Statistical analysis

Experiment 1 was run for 40 sessions; data from the first 5 and last four sessions were not included in the analysis because of uneven days of exposure to the rewarded images. Thus, there were a total of 31 sets of rewarded visual images (image set), each with 5 d of exposure. Experiment 2 was run for 15 sessions using naturalistic visual images. For both experiments, RTs from attentional capture trials were analyzed using a repeated-measures ANOVA. Overall RT effects that differed by monkey or side were controlled for by random effects factors in the ANOVA models. All analyses were performed using custom software written in MATLAB (The MathWorks; RRID:SCR_001622).

In Experiment 1, to determine whether subjects learned the value of rewarded images during Acquisition, pupil size was recorded for the duration of each trial. Percent change in pupil size during passive viewing of the image was measured relative to a baseline period extending from 250 ms before to 250 ms after image onset on each trial. This period was chosen because pupil stabilization (after fixation) had been achieved by the start of the baseline window, and because the pupil response typically lags image onset by 250 ms or more (Mitz et al., 2017). Pupil responses were grouped by the day in which the image was introduced, leading to pupil measurements on the first day images were introduced (day 1), and after 2, 3, 4, and 5 d of cumulative exposure. Data were resampled using 25-ms time bins and plotted as a function of days of exposure, also referred to as “days since novel.” The pupil response because of the initial light reflex was considered to be established by 750 ms after image onset. Data from 751-1250 ms were taken to represent the pupil response during restricted viewing of the image. The pupil response during this time period was averaged within value category (high, low) on a given day of exposure (1-5) to obtain a mean pupil response. Percent change in pupil size relative to baseline was analyzed using a nested mixed-effects ANOVA. Fixed factors included group (lesion, control), monkey (1-3), day since novel (1-5, continuous), and image value (high, low). Image set (1-31, random) was nested under monkey, and monkey was nested under group. Responses to overtrained images were analyzed separately. For individual monkeys, the average change in pupil size was collapsed across days. The pupil responses for high- and low-value images were compared using paired sample *t* tests.

RTs for the Attentional Capture block in Experiment 1 were defined as the time between image onset and the initiation of the saccade that finished in the circular peripheral target window. *Post hoc* analysis eliminated trials containing more than one saccade and/or those that did not originate from within a 3 DVA circular window. Initially, monkeys were allowed 300 ms to saccade to the peripheral target, but this time was increased to 1000 ms so as to not artificially constrain RTs and potentially affect our dependent measure. This change did not significantly affect RTs (*F*_(1,3703)_ = 0.46, *p* = 0.50, ANOVA). An average RT was calculated for each image per side per day and organized relative to the day the image was first introduced (as described above for pupil response). Images that did not have enough RTs from both sides because of random presentation or *post hoc* elimination (i.e., saccade origination location, number of saccades) were removed from the ANOVA. RTs to overtrained images were not included. RTs (log-transformed, dependent variable) were analyzed using a nested mixed-effects ANOVA that specified group (lesion, control), monkey (1-3), days since novel (1-5, continuous), image value (high, low), allowed RT (300 ms, 1000 ms), and side (left, right) as fixed factors and image set (1-31) as a random effect. Monkey was nested below group, and image set was nested below monkey.

As in Experiment 1, RTs for the Attentional Capture block of Experiment 2 were defined as the time between image onset and the initiation of the saccade that finished in the peripheral target window. *Post hoc* analyses of saccades were identical to those described for Experiment 1. An average RT per day was calculated for each image type and target side and analyzed using nested mixed-effects ANOVA models. To test the effect of intact images relative to scrambled images on RT (log-transformed RT, dependent variable), a mixed-effects ANOVA model was used with factors, including group (lesion, control), monkey (1-3), image type (intact, scrambled), and target side (left, right) as fixed factors and session number (1-15) as a random effect. Monkey was nested under group and session was nested under monkey. To determine how RT (log-transformed RT, dependent variable) differed among scrambled image types, a mixed-effects ANOVA model was used. Factors included group (lesion, control), monkey (1-3), image type (scrambled conspecific images, scrambled predator images, scrambled neutral object images), and target side (left, right) as fixed factors and session number (1-15) as a random effect. Session number was nested under monkey, and monkey was nested under group. To determine how RT (log-transformed RT, dependent variable) differed among intact image types, we used the same mixed-effects ANOVA model as for scrambled images but applied to data from intact images. To test the difference of RT (log-transformed RT, dependent variable) between groups for image types (conspecific, predator, and neutral), three separate mixed-effects ANOVA models were used, one for each image type. Fixed effects factors included group (lesion, control), monkey (1-3), and target side (left, right) while session number (1-15) was a random effect nested under monkey which was nested under group.

## Results

Three monkeys with selective, bilateral lesions of the amygdala (Fig. 1) and three unoperated controls were studied to test the role of the amygdala in attentional capture by recently learned reward-predictive visual images (Experiment 1). For comparison, we also assessed attentional capture by naturalistic images with innate biological value in the same two groups of monkeys (Experiment 2). In Experiment 1, monkeys learned the value of images in Acquisition trials (Fig. 3*A*). After exposure to images predicting a high or low probability of reward, monkeys performed an Attentional Capture task using the recently learned reward-predictive stimuli (Fig. 3*B*). In this design, we expected that attentional capture would be reflected in a main effect of image value. That is, we predicted that intact monkeys would show a slower RT for high reward versus low reward images. In addition, we anticipated that monkeys with amygdala lesions would show a reduction in attentional capture by rewarded images, as evidenced by a significant interaction of group and image value. In Experiment 2, monkeys performed the same Attentional Capture task using images of neutral objects, as well as naturalistic images of conspecifics and predators, categories with innate biological value (Fig. 3*C*). Based on past work, we predicted that controls would show greater attentional capture for the conspecific and predator images relative to neutral images. In addition, we expected that, relative to the unoperated controls, monkeys with amygdala lesions would show a reduction in attentional capture by images in both conspecific and predator categories, as evidenced by a significant interaction of group and image type.

### Experiment 1: attentional capture by rewarded images

#### Acquisition of stimulus-reward associations

**Figure 4.**
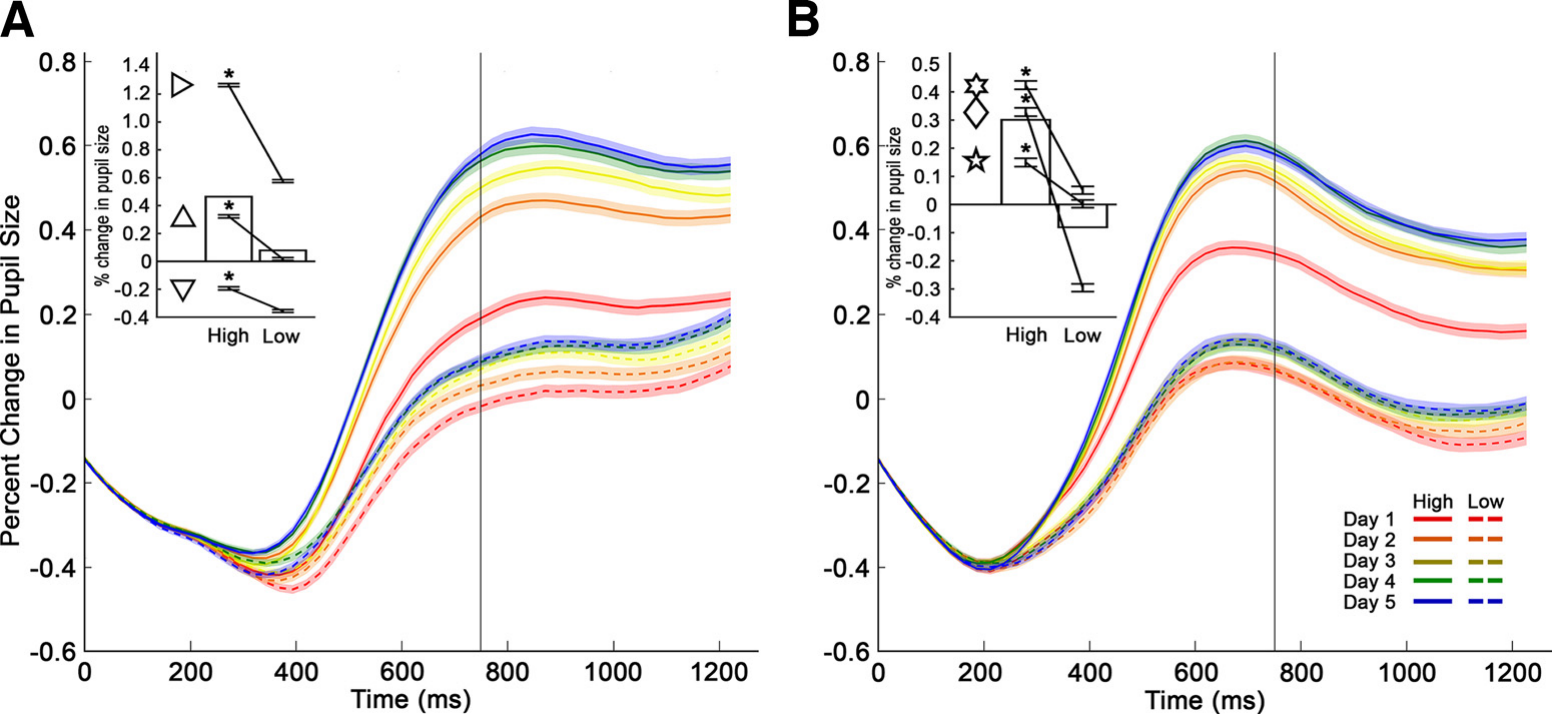
Conditioned pupil response during acquisition. ***A***, Traces represent the average percent change in pupil size relative to the baseline period for the 3 monkeys with amygdala lesions, plotted as a function of reward value (high, low) and days since novel (1-5). Solid lines indicate pupil responses to high-value images. Dashed lines indicate responses to low-value images. Shaded regions represent SEM. ***B***, Traces represent the average percent change in pupil size relative to the baseline period for the 3 unoperated control monkeys. Conventions are the same as in ***A***. Conditioned pupil responses were taken to be between 751 and 1250 ms (represented by the traces to the right of the vertical black line at 750 ms). Both groups of monkeys displayed greater pupil size changes for images predicting a high probability of reward relative to those predicting a low probability of reward, indicating that the monkeys had learned the image values. Insets, Bars represent the average change in pupil size for both high and low reward value images collapsed across days. Lines indicate the average pupil response (mean ± SEM) for individual monkeys (L1-L3 for the lesion group [***A***] and C1-C3 for the control group [***B***]). Ordinates of insets use different scales. Symbols represent scores of individual monkeys: inverted triangle, L1; sideways triangle, L2; upright triangle, L3; diamond, C1; 5 point star, C2; 6 point star, C3. *p < 0.01 (paired sample *t* test).

Change in pupil size during passive viewing of images in each session's Acquisition block served as our measure of stimulus-reward learning. Pupil responses were grouped by reward probability (high, low) and number of days since novel (1-5). As shown in Figure 4, all monkeys learned the high- and low-value images; they showed a greater average change in pupil size for high-value images compared with low-value images. A nested mixed-effects ANOVA (see Statistical analysis) revealed main effects of image value (*F*_(1,1859)_ = 18.04, *p* = 2.8730 × 10^−5^) and days since novel (*F*_(1,1859)_ = 44.85, *p* = 1.6177 × 10^−10^), findings consistent with learning. There was also a main effect of group, indicating that the two groups had different overall changes in pupil size (*F*_(1,1859)_ = 9.100, *p* = 0.003). As can be appreciated by comparing the insets in Figure 4*A*, *B*, the change in pupil size in response to passive viewing of both the high and low probability of reward stimuli is, on average, greater for monkeys in the amygdala lesion group. This group effect is likely because of the more sustained pupil diameter change in the amygdala lesion group relative to controls. Finally, pupil size did not differ as a function of image set (i.e., the collection of images shown to monkeys on a given day as image set 1, set 2, etc., and included in the ANOVA at any given time), suggesting that there was no inherent effect on pupil size carried by individual images (*F*_(180,1859)_ = 0.933, *p* = 0.68).

*Post hoc* tests indicated that each monkey with an amygdala lesion (L1: *t*_(174)_ = 2.70, *p* = 0.008; L2: *t*_(174)_ = 19.07, *p* = 1.72 × 10^−44^; L3: *t*_(174)_ = 9.63, *p* = 1.15 × 10^−18^, paired *t* test, Fig. 4*A*, inset) and each unoperated control (C1: *t*_(174)_ = 15.12, *p* = 1.58 × 10^−33^; C2: *t*_(174)_ = 4.44, *p* = 1.61 × 10^−5^; C3: *t*_(174)_ = 6.31, *p* = 2.27 × 10^−9^, paired *t* test, Fig. 4*B*, inset) exhibited a greater change in pupil size for high-value images compared with low-value images.

In addition to responding to changes in luminance and focal distance, pupil diameter reflects at least three categories of stimulus processing and/or attention: alerting, orienting to salient stimuli, and cognition (Bradley, 2009; Joshi and Gold, 2020; Strauch et al., 2022). For example, pupil diameter is known to increase during problem solving (Hess and Polt, 1964) and during decision-making under uncertainty (Lempert et al., 2015; Urai et al., 2017). Here, because the cognitive demands of the Acquisition trials were identical across reward levels, and because we controlled for luminance, we ascribe changes in pupil diameter that accrue during the Acquisition trials to learned changes in the salience of stimuli.

#### Amygdala lesions fail to diminish attentional capture by rewarded images

**Figure 5.**
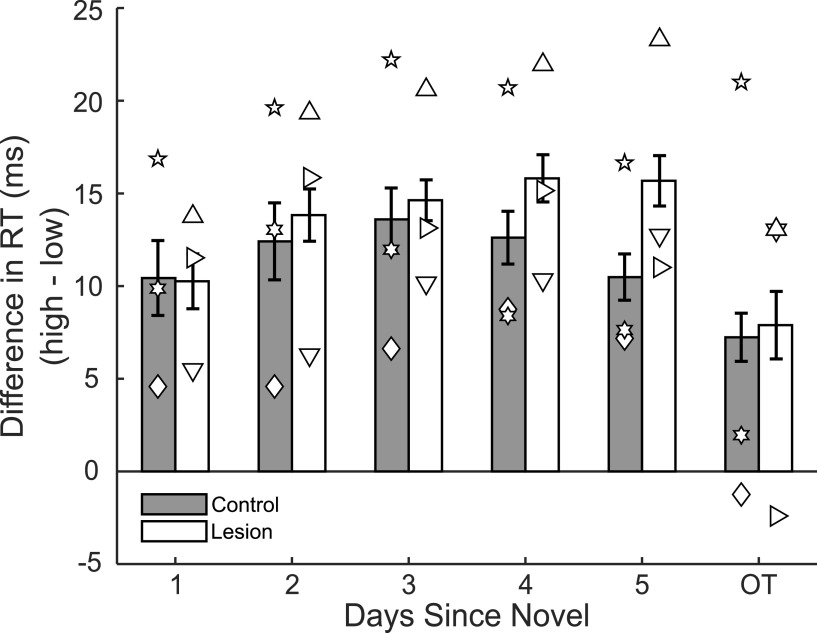
Rewarded images capture attention, and amygdala lesions do not disrupt this effect. Bars represent the difference (high minus low probability of reward images) in RTs (mean ± SEM) to initiate a saccade to a peripheral target as a function of days since novel for unoperated controls (gray bars) and monkeys with amygdala lesions (white bars). Symbols represent scores of individual monkeys: inverted triangle, L1; sideways triangle, L2; upright triangle, L3; diamond, C1; 5 point star, C2; 6 point star, C3.

Given that all monkeys successfully acquired the image-reward associations, a prerequisite for asking our question about the neural basis for attentional capture, we evaluated our dependent measure on the attentional capture trials: RT to initiate a saccade to the peripheral target when a rewarded image was simultaneously presented at fixation. As shown in Table 1, RT differed by group; monkeys with amygdala lesions were slower than controls to initiate saccades to the peripheral target (*F*_(1,3703)_ = 117.85, *p* = 3.81 × 10^−24^). There was a significant interaction of group and days since novel (*F*_(1,3703)_ = 7.31, *p* = 0.007), possibly indicating that monkeys with amygdala lesions learned the image-value assignments at a different rate from the controls (*F*_(1,3703)_ = 90.33, *p* = 3.59 × 10^−19^). In addition, there was a significant main effect of days since novel; RTs decreased with increased exposure to image sets (*F*_(1,3703)_ = 90.33, *p* = 3.59 × 10^−19^). Contrary to our prediction, however, there was no interaction of group and image value (*F*_(1,3703)_ = 0.812, *p* = 0.369). Both groups exhibited longer RTs for images of high value compared with low value (*F*_(1,3703)_ = 26.25, *p* = 9.18 × 10^−7^), as evident in the positive differences (high minus low) depicted in Figure 5. RTs did not differ between any of the 31 image sets (*F*_(108,3703)_ = 1.17, *p* = 0.15, ANOVA), nor did they differ when the peripheral target was presented on either the left or the right (*F*_(1,3703)_ = 3.29, *p* = 0.07, ANOVA).

**Table 1.** Mean RTs in attentional capture task (Experiment 1)*^a^*

Subject	Image value	1	2	3	4	5	Mean days 1-5
L1	High	180.8	176.8	177.9	176.0	176.6	177.6
	Low	175.3	170.5	167.7	165.7	163.9	168.6
L2	High	188.1	184.9	179.7	179.5	176.7	181.8
	Low	176.6	169.0	166.5	164.4	165.7	168.4
L3	High	194.7	196.2	196.3	197.8	198.0	196.6
	Low	181.0	176.9	175.7	175.8	174.7	176.8
C1	High	151.2	149.2	149.9	149.9	148.9	149.8
	Low	146.7	144.6	143.2	141.1	141.7	143.5
C2	High	178.4	175.8	174.8	171.7	166.5	173.4
	Low	161.5	156.2	152.6	150.9	149.8	154.2
C3	High	189.1	184.8	178.7	174.3	172.0	179.8
	Low	179.2	171.8	166.8	165.9	164.4	169.6

*^a^*Mean RTs (ms) for each monkey to initiate a saccade to the peripheral target on trials in which the image presented at fixation had been associated with a high probability of reward (High) or a low probability of reward (Low). L1-L3, monkeys with bilateral excitotoxic lesions of the amygdala; C1-C3, unoperated controls. Numerals 1-5: number of days images had been paired with reward (days since novel). Mean days 1-5: mean of RTs across days 1-5.

In sum, in the Acquisition phase, both controls and monkeys with amygdala lesions learned stimulus-reward-value associations. In addition, all monkeys showed a robust effect of image reward value on attentional capture, evidenced by a slower initiation of a saccade to the peripheral target when presented with a high-value relative to low-value image at fixation. Contrary to our prediction, however, there was no indication that amygdala removals reduced attentional capture by rewarded images.

### Experiment 2: attentional capture by predator and conspecific images

#### Amygdala lesions reduce attentional capture by naturalistic images with innate biological value

**Figure 6.**
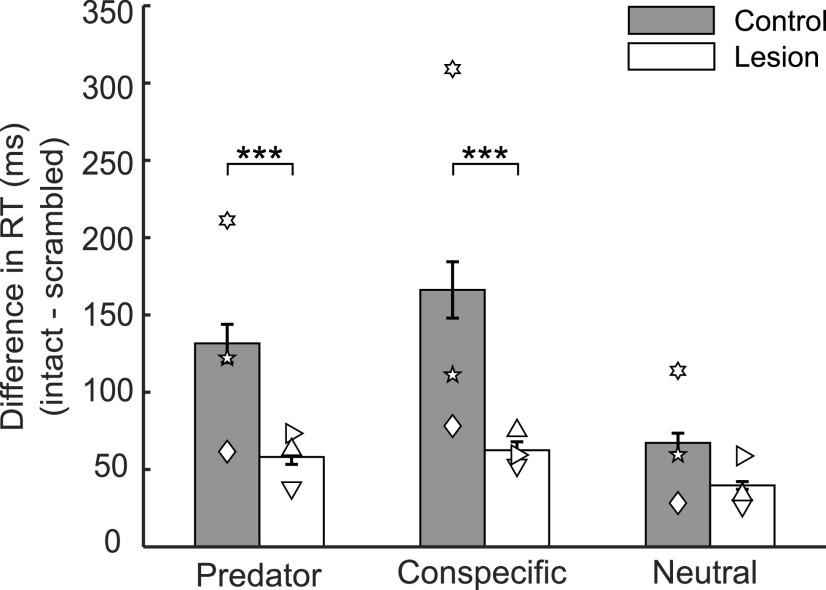
Naturalistic images of predators and conspecifics capture attention, and amygdala lesions significantly attenuate this effect. Bars represent the difference (intact minus matched scrambled image) in RTs (mean ± SEM) to initiate a saccade to a peripheral target as a function of image type for unoperated controls (gray bars) and monkeys with amygdala lesions (white bars). ***p < 0.001 (mixed effect ANOVA). Symbols represent scores of individual monkeys: inverted triangle, L1; sideways triangle, L2; upright triangle, L3; diamond, C1; 5 point star, C2; 6 point star, C3.

In Experiment 2, we evaluated attentional capture by intact and scrambled images of conspecifics and predators, using neutral images (intact and scrambled) as a control. Differences in RTs (intact minus scrambled) can be seen in Figure 6 for both groups as a function of image type. An ANOVA on RTs with factors of group (lesion, control), monkey (1-3), image type (intact, scrambled), and target side (left, right) revealed that RTs for intact images were longer than for scrambled images (*F*_(1,1075)_ = 1.35 × 10^3^, *p* = 1.35 × 10^−53^) as evident in the positive differences (intact minus scrambled) depicted in Figure 6. Next, separate ANOVAs were conducted for intact and scrambled images. Monkeys showed no differences in RTs when scrambled images were presented on the screen (*F*_(1,355)_ = 1.08, *p* = 0.30), and there was no interaction of group by image type (*F*_(4,355)_ = 1.21, *p* = 0.31). When intact images were presented, however, there was a significant group by image type interaction (*F*_(2,539)_ = 15.03, *p* = 9.85 × 10^−7^), indicating that monkeys with amygdala lesions had altered RTs relative to controls for some image types. To examine the group differences in further detail, individual ANOVAs were run within each image type. Although no group difference in RT was found for neutral images (*F*_(1,179)_ = 0.30, *p* = 0.59), group differences emerged for both predator images (*F*_(1,179)_ = 13.32, *p* = 4.55 × 10^−4^) and conspecific images (*F*_(1,179)_ = 34.22, *p* = 9.19 × 10^−8^). In each case, the group differences emerged because monkeys with amygdala lesions were faster than controls to initiate a saccade to the peripheral target. Thus, as expected based on earlier work using other paradigms, amygdala lesions disrupted attentional capture by naturalistic images of conspecifics and predators.

The RT differences in Experiment 2 were greater than the RT differences in Experiment 1. Whereas the RT contrast in Experiment 2 was between predator/conspecific images and scrambled images, the RT contrast in Experiment 1 was between two valuable items: high and low probability of reward images. We intentionally used high versus low probability of reward to gain better control of any effects on attentional capture of learning and magnitude of reward. If we had used high reward versus no reward, or high reward versus scrambled images, we expect we would have observed larger RT differences in Experiment 1.

## Discussion

To test the causal contributions of the amygdala to allocating attention, we used the attentional capture task, a sensitive, quantitative assay of attentional bias. We examined the role of the amygdala in attentional capture by rewarded images (Experiment 1) and by conspecific and predator images (Experiment 2). In Experiment 1, we found that all monkeys, controls and operated alike, showed longer RTs when they were presented with high-value images, with the effect of image value being equivalent for both groups. Thus, our monkeys showed robust attentional capture by rewarded images, and there was no influence of amygdala removal on this effect. In Experiment 2, we found that the groups differed when they were presented with conspecific or predator images, but not neutral images. Specifically, relative to controls, monkeys with amygdala lesions showed faster RTs when either conspecific or predator images were presented at fixation. Our results suggest two conclusions: (1) the amygdala makes a causal contribution to directing attention to conspecifics and predators; and (2) even when damage to the amygdala is complete, or nearly so, there remains a capability to direct attention to valuable objects in the environment.

### Amygdala, attention, and reward

The pending availability of reward has been shown to improve performance; lowering error rates and hastening responding when rewarded stimuli are part of a goal-directed task (Bowman et al., 1996; Hollerman et al., 1998; Kobayashi et al., 2002; Roesch and Olson, 2003; Stănişor et al., 2013). These findings are consistent with a body of literature demonstrating that stimuli associated with reward capture attention in both monkeys (Bell et al., 2004; Fecteau et al., 2004; Peck et al., 2009, 2013; Yasuda et al., 2012; Foley et al., 2014; Peck and Salzman, 2014a,b) and humans (Anderson et al., 2011a; Chelazzi et al., 2013; Failing and Theeuwes, 2014; Le Pelley et al., 2016, 2019; Bucker and Theeuwes, 2017; Wang et al., 2018; Kim and Anderson, 2019a,b). Monkeys with amygdala lesions are known to show deficits in perceiving, learning, or acting on reward predictive stimuli (Baxter and Murray, 2002; Rygula et al., 2015; Costa et al., 2016). Further, neurophysiological recordings in monkeys provide evidence that neurons in the amygdala are modulated by attention to either rewarding (Peck et al., 2013; Peck and Salzman, 2014b) or aversive (Peck and Salzman, 2014a) visual stimuli. Yet few studies have investigated the role of the amygdala in attention per se. These neurophysiological findings, together with the finding that the amygdala is involved in allocating attention to images of conspecific faces (Dal Monte et al., 2015; Taubert et al., 2018), led us to predict that the amygdala would also be essential for allocating attention to images of reward-predictive stimuli.

In the present study, in both monkeys with amygdala lesions and controls, goal-directed performance in the attentional capture task was negatively impacted by the presence of reward-predictive images. Thus, the amygdala is not necessary for allocating attention to reward-predictive stimuli. Given that the same monkeys with amygdala lesions showed reduced attentional capture by other categories of images, the negative results with rewarded images cannot be ascribed to ineffective amygdala lesions, lack of sensitivity of the task, or inappropriate task parameters.

There are at least three issues requiring explanation. First, given that the experience with rewarded images accrued across days, we expected monkeys to have greater difficulty avoiding distractors with increased exposure and, therefore, to exhibit slower RT across days. Contrary to this idea, however, monkeys showed a faster RT across days. Perhaps greater image familiarity allowed monkeys to better suppress the tendency to gaze at rewarded images. The present study was not designed to dissect the possible sources of attentional bias, however, so this explanation must remain speculative.

Second, monkeys with amygdala lesions showed overall larger changes in pupil sizes during acquisition of the stimulus-reward associations and overall slower RTs during attentional capture trials relative to controls (Fig. 4; Table 1). Because all behavioral assessments were conducted concurrently, differences in the manner in which the task was run, in the parameters used, or the like, are unlikely to account for group differences. One effect of amygdala lesions is to increase heart rate (Mitz et al., 2017), suggesting that amygdala lesions increase sympathetic tone. If so, the group difference in sympathetic tone might explain both the larger changes in pupil size and the slowed RT. In support of this idea, amygdala lesions in macaques have been found to alter the impact of arousal on RTs (Fujimoto et al., 2021). In intact monkeys, greater arousal is associated with shorter RTs. After amygdala lesions, however, greater arousal is associated with longer RTs.

A third point that requires explanation is the neural basis for attentional bias to rewarded images. If the amygdala is not essential for allocating attention to rewarded stimuli, what neural structures support the process? The frontal eye fields, superior colliculus, and parietal cortex are known to support allocation of attention (Moore and Zirnsak, 2017). Although most evidence indicates a role for these areas in allocation of spatial attention, some work points to a role for a ventral prearcuate area, near the frontal eye fields, in feature-based attention (Bichot et al., 2015), and for a small region of cortex on the floor of the superior temporal sulcus in both spatial and nonspatial attention (Bogadhi et al., 2019, 2021). Finally, neurons in both the head and tail of the caudate nucleus encode flexible and stable object values, respectively, and operate as part of a circuit projecting via the substantia nigra pars reticulata to the superior colliculus to facilitate gaze toward valuable objects (Kunimatsu et al., 2019; Amita et al., 2020).

Because our study examined amygdala involvement in attentional bias for image-reward associations learned postoperatively, we cannot rule out the possibility that the amygdala would play a role if we had examined the influence of amygdala lesions on images learned preoperatively, or examined the influence of temporary inactivations of the amygdala. In addition, we cannot rule out the possibility that, had we used even more highly rewarded images, or assessed RT for rewarded versus scrambled images, we might have observed an effect of amygdala lesions on attentional capture. Future studies should assess these possibilities.

### Amygdala, attention, and conspecifics

Several findings suggest that macaques have an innate interest in images of conspecifics. The preferential viewing of faces over objects (Taubert et al., 2017) and prioritization of faces for viewing (Taubert et al., 2017) is likely innate, as visual tracking of faces occurs early in life (Farroni et al., 2005). Prioritization of viewing conspecifics is also revealed in paradigms pitting reward procurement against viewing of conspecific images (Deaner et al., 2005; Rudebeck et al., 2006). In these settings, amygdala lesions or amygdala disconnection from PFC disrupt the tendency to view faces (Taubert et al., 2018; Pujara et al., 2022). Our finding that images of conspecifics produce attentional capture complement those of Dal Monte et al. (2015), who used a similar paradigm to study attentional capture by faces (specifically, images of face parts, including eyes, nose, and mouth) of macaque monkeys. Our results extend those findings by showing that attentional capture by socially relevant information extends beyond faces to include female perinea. Other features relevant to primate social behavior may well have a similar status.

### Amygdala, attention, and predators

Primates, including macaques and humans, often exhibit strong reactions to predators, especially snakes, and snake-naive macaques have been found to exhibit defensive responses to snakes (Nelson et al., 2003), suggesting that the responses are innate. In line with findings documenting a lack of defensive responses to snakes following amygdala lesions in adult (Kalin et al., 2001; Meunier and Bachevalier, 2002; Izquierdo et al., 2005; Chudasama et al., 2009) and infant macaques (Bliss-Moreau et al., 2011; Raper et al., 2019; Medina et al., 2020), we found that the amygdala is necessary for allocating attention to naturalistic images of predators. The failure of monkeys with amygdala lesions to marshal defensive responses to snakes (Izquierdo et al., 2005) and the reduced attentional capture by predator images presumably reflect the loss of amygdala circuits that activate species-specific defensive and orienting reactions.

It has been proposed that a “fast pathway” from the superior colliculus to the amygdala via the pulvinar supports defensive responses to snakes (LeDoux, 1994). A role for the superior colliculus in generating defensive responses to snakes has been reported in New World monkeys (Maior et al., 2011). Against the idea of a “fast pathway,” however, no directly connecting pathway from the superior colliculus to pulvinar to amygdala has been demonstrated. The best evidence to date is that a given region of the pulvinar both receives projections from the superior colliculus and gives rise to projections to the amygdala (Elorette et al., 2018). Given that the activity of neurons in the central nucleus of the amygdala influences eye movements in motivational contexts (Maeda et al., 2020), future studies should investigate the role of the primate central nucleus of the amygdala in gaze bias to faces and predators.
